# Restoration of FVIII Function and Phenotypic Rescue in Hemophilia A Mice by Transplantation of MSCs Derived From *F8*-Modified iPSCs

**DOI:** 10.3389/fcell.2021.630353

**Published:** 2021-02-11

**Authors:** Liyan Qiu, Mi Xie, Miaojin Zhou, Xionghao Liu, Zhiqing Hu, Lingqian Wu

**Affiliations:** Center for Medical Genetics & Hunan Key Laboratory of Medical Genetic, School of Life Sciences, Central South University, Changsha, China

**Keywords:** hemophilia A, iPSCs, gene correction, ribosomal DNA, MSCs, intravenous injection

## Abstract

Hemophilia A (HA), an X-linked recessive congenital bleeding disorder, affects 80%–85% of patients with hemophilia. Nearly half of severe cases of hemophilia are caused by a 0.6-Mb genomic inversion (Inv22) that disrupts *F8*. Although viral-based gene therapy has shown therapeutic effects for hemophilia B (HB), this promising approach is not applicable for HA at the present stage; this limitation is mainly due to the large size of *F8* cDNA, which far exceeds the adeno-associated virus (AAV) packaging capacity. We previously reported an *in situ* genetic correction of Inv22 in HA patient-specific induced pluripotent stem cells (HA-iPSCs) by using TALENs. We also investigated an alternative strategy for targeted gene addition, in which cDNA of the B-domain deleted *F8* (*BDDF8*) was targeted at the rDNA locus of HA-iPSCs using TALENickases to restore FVIII function. Mesenchymal stem cells (MSCs) have low immunogenicity and can secrete FVIII under physiological conditions; in this study, MSCs were differentiated from *F8*-corrected iPSCs, *BDDF8*-iPSCs, and HA-iPSCs. Differentiated MSCs were characterized, and FVIII expression efficacy in MSCs was verified *in vitro*. The three types of MSCs were introduced into HA mice *via* intravenous injection. Long-term engraftment with restoration of FVIII function and phenotypic rescue was observed in HA mice transplanted with *F8*-corrected iMSCs and *BDDF8*-iMSCs. Our findings suggest that *ex vivo* gene therapy using iMSCs derived from *F8*-modified iPSCs can be feasible, effective, and promising for the clinical translation of therapeutic gene editing of HA and other genetic birth defects, particularly those that involve large sequence variants.

## Introduction

Hemophilia A (HA) is an X-linked recessive congenital bleeding disorder caused by mutations in the factor VIII (FVIII) gene (*F8*) that leads to deficient blood coagulation. Spontaneous or traumatic bleeding in daily life or during surgery is the main symptom in HA patients, and the incidence is one in 5,000 male births (Nienhuis et al., 2017). Severe HA patients have only 1% or less of normal plasma FVIII activity. Currently, the main clinical treatment of HA is preventive or on-demand intravenous injections of FVIII concentrates from normal human plasma or recombinant FVIII protein. However, due to the short half-life of FVIII, patients require repeated injections, which increase the risks of infection and induce FVIII inhibitors (Berntorp and Shapiro, 2012; Weyand and Pipe, 2019).

Hemophilia is a monogenic disease considered suitable for gene therapy because a slight increase in plasma coagulation factor activity can lessen the bleeding phenotype (Graw et al., 2005; Perrin et al., 2019). Gene therapy of hemophilia based on viral vectors has been extensively investigated. Adeno-associated virus (AAV)-mediated gene therapy of hemophilia B (HB) produced promising progress in HB animal models (Crudele et al., 2015) and clinical trials (George et al., 2017; Miesbach and Sawyer, 2018). However, certain challenges of AAV-based gene therapy for HA remain mainly because the size of the FVIII coding sequence is too large (7 kb) to be packed into the AAV vector. Therefore, a smaller B-domain-deleted human *F8* (*BDDF8*) cDNA of 4.4 kb was incorporated in the AAV vector to obtain therapeutic effects in animal models (Sabatino et al., 2011; McIntosh et al., 2013) and in clinical trials (Rangarajan et al., 2017; Batty and Lillicrap, 2019; Pasi et al., 2020).

Cell-based gene therapy has been actively studied as a potential alternative treatment for HA. Transplanted cells can provide sustained production of FVIII *in vivo*. Since endothelial cells (ECs) (Kumaran et al., 2005; Follenzi et al., 2008) and mesenchymal stem cells (MSCs) (Follenzi et al., 2012; Sanada et al., 2013) can physiologically secrete functional FVIII protein, these two cell types are generally the preferred target cells for HA gene therapy. Several studies have demonstrated that transplanted liver sinusoidal endothelial cells (LSECs) or ECs can synthesize FVIII in the liver and correct the bleeding phenotype in HA mice (Follenzi et al., 2008; Shi et al., 2010; Yadav et al., 2012; Fomin et al., 2013). However, LSECs and ECs are not readily available and have poor proliferation ability. MSCs have low immunogenicity and strong renewal ability and are easy to separate and propagate *in vitro*. Various sources of MSCs were modified using viral vectors *in vitro* and then transplanted into HA mice to restore the FVIII function. Some studies have used bone marrow-derived MSCs to achieve phenotypic correction in mice (Follenzi et al., 2012). Other studies have focused on long-term therapeutic plasma levels of FVIII in HA mice by systemic delivery of MSCs derived from *hFVIIIBD* transgenic mice (Wang et al., 2013) or by transplantation of virus-modified MSCs *in utero* (Kumar et al., 2018).

Due to the self-renewal potential and multidirectional differentiation, human induced pluripotent stem cells (iPSCs) can be used as an applicable autologous cell source for *ex vivo* gene therapy for HA (Takahashi and Yamanaka, 2006; Wong and Chiu, 2011). Non-viral genome-editing approaches in iPSCs include actively developing *in situ* gene correction at the *F8* locus (Park et al., 2015; Wu et al., 2016; Hu et al., 2019) and ectopic integration of *BDDF8* at the safe harbor loci, such as the ribosomal DNA (rDNA) locus (Pang et al., 2016) and the H11 locus (Park et al., 2019). Differentiation of iPSCs into MSCs can be an efficient means to generate many batches of MSCs. On the one hand, iPSC-derived MSCs and bone marrow-derived MSCs have similar cell characteristics and differentiation potential (Diederichs and Tuan, 2014). On the other hand, iPSCs can be genetically modified and then continuously differentiated into MSCs to maintain long-term therapeutic gene expression for autologous gene therapy.

In our previous studies, cells from urine of a patient with severe HA with *F8* intron 22 inversion (Inv22) were collected, reprogrammed into HA patient-specific iPSCs (HA-iPSCs), and used to generate *F8*-corrected iPSCs *via in situ* gene correction using TALENs (Wu et al., 2016); *BDDF8*-iPSCs were generated by targeted integration of *BDDF8* at the rDNA locus using TALENickases (Pang et al., 2016). In the present study, *F8*-corrected iPSCs, *BDDF8*-iPSCs, and HA-iPSCs were differentiated into iMSCs (iPSCs-derived MSCs), which were characterized and transplanted into HA mice by intravenous injection. Functional restoration of FVIII and phenotypic rescue were achieved in HA mice transplanted with iMSCs derived from *F8*-corrected iPSCs and *BDDF8*-iPSCs. Our data demonstrate that *ex vivo* therapeutic gene editing *via F8*-modified iPSC-derived iMSCs is feasible, effective, and promising for the clinical translation of HA gene therapy.

## Materials and Methods

### Cell Culture

Normal human iPSCs (DYR0100) were purchased from ATCC. HA-iPSCs, 17-9-iPSCs (*F8*-corrected iPSCs) (Wu et al., 2016), and T-7-iPSCs (*BDDF8*-iPSCs) (Pang et al., 2016) were generated previously by our group. Briefly, *F8*-corrected iPSCs were obtained by targeting the coding sequence of *F8* exons 23–26 at the junction of exon 22 and intron 22 in HA-iPSCs. *BDDF8*-iPSCs were obtained by site-specific integration of *BDDF8* at the rDNA locus in HA-iPSCs. All iPSCs were routinely cultured (37°C, 5% CO_2_) on Matrigel (BD Biosciences, United States)-coated 12-well plates (Corning, United States) in mTesR1 medium (STEMCELL Technologies, Canada). iMSCs derived from iPSCs were routinely cultured (37°C, 5% CO_2_) on 0.1% gelatin (STEMCELL Technologies)-coated dishes in MSC medium containing DMEM/LG (HyClone, United States) supplemented with 10% FBS (Gibco, United States), 2 mM GlutaMAX^TM^ (Gibco), and 0.1% bFGF (Gibco).

### Derivation of iMSCs From iPSCs

A STEMdiff^TM^ mesenchymal progenitor kit (STEMCELL Technologies) was used to differentiate normal human iPSCs (hiPSCs), HA-iPSCs, 17-9-iPSCs, and T-7-iPSCs into hiMSCs, HA-iMSCs, 17-9-iMSCs, and T-7-iMSCs, respectively. According to the manufacturer’s protocol, iPSCs were seeded on a Matrigel-coated 12-well plate at 5 × 10^4^ cells/cm^2^ in mTesR1 containing 10 μM Y27632. The cells were cultured in mTesR1 for 2 days and in STEMdiff^TM^-ACF mesenchymal induction medium for 4 days. The cells were then cultured in STEMdiff^TM^-ACF medium for additional 2 days and passaged on a 6-well plate precoated with STEMdiff^TM^-ACF attachment substrate. The cells were passaged every 3 days. After three passages, the cells were seeded onto a 0.1% gelatin-coated 10-cm dish in MSC medium. The medium was changed every day during differentiation.

### Characterization of iMSCs

iMSCs (iPSCs-derived MSCs) were suspended at a concentration of 1 × 10^5^ cells/mL in 1 × DPBS. A total of 5 × 10^4^ cells were incubated with BB515-conjugated CD44, Precp-Cy5.5-conjugated CD73, PE-Cy7-conjugated anti-human CD90, APC-conjugated CD105, BV421-conjugated anti-human CD34, CD45, and HLA-DR (BD Biosciences) at room temperature for 30 min in the dark. Then, the stained cells were washed twice in DPBS. Flow cytometry analysis was performed by a flow cytometer (BD Biosciences) to detect the expression of the cell surface molecules of differentiated iMSCs.

### Identification of Differentiation Potential of iMSCs

The differentiation potential of iMSCs was identified by osteogenesis (Gibco), adipogenesis (STEMCELL Technologies), and chondrogenesis (STEMCELL Technologies) differentiation kits according to the manufacturer’s protocols.

### RT-PCR and qRT-PCR

Total RNA was extracted using TRIzol reagent (Sigma-Aldrich, United States), and RNA was treated with gDNA wiper mix (Vazyme, China) to eliminate genomic DNA. Then, a 500 ng RNA sample was reverse transcribed using Hiscrip^®^ II Q RT Supermix (Vazyme). Primers based on exons 19 and 23 were used to detect the transcript of *F8*. Primers based on exons 23 and 26 were used to detect relative *F8* (exons 23–26) mRNA levels. The glyceraldehyde-3-phosphate dehydrogenase (GAPDH) gene was used as an endogenous control.

### FVIII Assay of Culture Supernatants and Cell Lysates

The culture supernatants were harvested in triplicate from 6-well plates 24 h after the medium was changed. The cells were dissociated with TrypLE^TM^ select enzyme (Gibco) and counted. After washing with DPBS, cell pellets were resuspended in 500 μL of sample diluent for ELISA (Cedarlane, Burlington, ON, Canada) and lysed by three freeze-thaw cycles. ELISA was performed using Paired Antibodies for ELISA-factor VIII:C (Cedarlane) according to the manufacturer’s instructions.

### Western Blot

Cell lysates of iPSCs and iMSCs were prepared using RIPA lysis buffer (Beyotime, China) supplemented with 1 × phenylmethanesulfonyl fluoride (PMSF, 1 mM) and Protease Inhibitor Cocktail (Sigma-Aldrich). Protein in the samples was measured by a Pierce^TM^ BCA protein assay kit (Thermo Fisher Scientific, United States). Protein samples (15 μg) were loaded on an electrophoresis gel that was run for 2 h at 120 V, and the proteins were transferred to PVDF membranes (Millipore, United States). After blocking with 5% non-fat milk in 0.1% TBST (TBS containing 0.1% Tween-20), the membrane was incubated overnight with anti-LMAN1 antibodies (Abcam, England) at 4°C. The membrane was then incubated with HRP-labeled anti-rabbit IgG (Sigma-Aldrich) for 1 h at room temperature, and the signal was visualized using an ECL detection kit (Thermo Fisher Scientific). Then, the membrane was washed with stripping buffer and 0.1% TBST. After blocking, the membrane was incubated overnight with anti-β-actin antibodies (Sigma-Aldrich) at 4°C. The membrane was incubated with anti-mouse IgG (Sigma-Aldrich) for 1 h at room temperature, and the signal was visualized using an ECL detection kit (Thermo Fisher Scientific).

### Transplantation of iMSCs Into HA Mice

The use and care of animals complied with the guidelines of the Ethics Committee of the School of Life Sciences of Central South University.

HA mice (strain: B6; 129S4-*F8* tm1Kaz/J; Jackson Laboratory) at 6–8 weeks of age were used for cell transplantation. C57BL/6 mice at 6–8 weeks of age were used as wild-type controls. Each mouse was anesthetized with Avertin (Sigma-Aldrich) and then infused with 2 × 10^6^ CM-Dil-labeled iMSCs derived from HA-iPSCs, 17-9-iPSCs, T-7-iPSCs, or hiPSCs. Mouse plasma was collected for the assay of FVIII activity at four time points: 1, 2, 3, and 4 weeks after transplantation. HA mice without transplanted iMSCs served as a negative control. In brief, the mice were anesthetized, and then retro-orbital blood samples were collected to isolate plasma. FVIII activity was evaluated using calibration plasma for coagulation tests (Stago, France), and Compact Max (Stago, France) was used to determine activated partial thromboplastin time (aPTT). The main organs (heart, liver, spleen, lung, and kidney) from the treated and HA mice were harvested to detect CM-Dil-positive cells by immunofluorescence analysis.

### Tail-Bleeding Assay

A tail-clip challenge was carried out 1 week after transplantation. Mice were anesthetized, and the tail-bleeding assays were performed as described previously (Yadav et al., 2012; Chen et al., 2017; Neumeyer et al., 2019). Briefly, the distal part of the mouse tail with a diameter of 1.5 mm was sheared. The tail was then immediately immersed in a 50 mL Falcon tube containing isotonic saline prewarmed in a water bath to 37°C. Each mouse was monitored for 20 min to record the bleeding time. Firm pressure on the tail was applied for 1 min, and the survival rates for each group of mice were recorded in 2 days.

### Statistical Analysis

GraphPad Prism 5.0 was used for data analysis. Data were analyzed using ANOVA for more than two groups. The survival curves were analyzed using the log-rank test. All values are presented as the mean ± SEM.

## Results

### Generation and Characterization of iMSCs Derived From iPSCs

**FIGURE 1:**
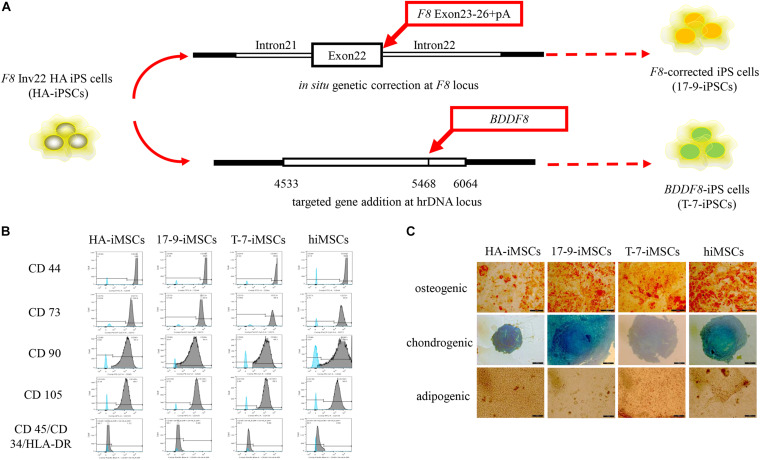
Derivation of iMSCs from iPSCs. **(A)** Schematic representation of the *in situ* genetic correction and targeted gene addition at the hrDNA locus in *F8* Inv22 HA iPS cells. **(B)** Flow cytometry analysis of surface markers in iMSCs derived from iPSCs. HA-iMSCs were derived from *F8* Inv22 HA iPS cells. 17-9-iMSCs were derived from *F8-*corrected iPS cells. T-7-iMSCs were derived from *BDDF8*-iPS cells. hiMSCs were derived from normal control hiPSCs. **(C)** Identification of osteogenic, chondrogenic, and adipogenic differential potential of iMSCs.

Our previous studies identified two forms of genetic modification in HA-iPSCs with Inv22 (Figure 1A). In this study, iPSCs with *in situ* genetic correction of *F8* (*F8*-corrected iPSCs) were named 17-9-iPSCs (Wu et al., 2016). iPSCs with ectopic integration of *BDDF8* at the rDNA locus (*BDDF8*-iPSCs) were named T-7-iPSCs (Pang et al., 2016). hiPSCs purchased from ATCC were used as a normal control. We differentiated HA-iPSCs, 17-9-iPSCs, T-7-iPSCs, and hiPSCs into HA-iMSCs, 17-9-iMSCs, T-7-iMSCs, and hiMSCs, respectively (Supplementary Figure 1A). iMSCs derived from iPSCs showed typical fibroblast-like morphology (Supplementary Figure 1B). All iMSC lines were positive for CD44, CD73, CD90, and CD105 and were negative for CD34, CD45, and HLA-DR (Figure 1B), which was consistent with a previous report (Xu et al., 2019). Multilineage potential tests showed that HA-iMSCs, 17-9-iMSCs, T-7-iMSCs, and hiMSCs have osteogenic, chondrogenic, and adipocytic differentiation abilities (Figure 1C). These results indicated that iPSCs were successfully and efficiently differentiated into iMSCs.

### Verification of FVIII Expression in *F8*-Modified iPSCs and iMSCs

**FIGURE 2:**
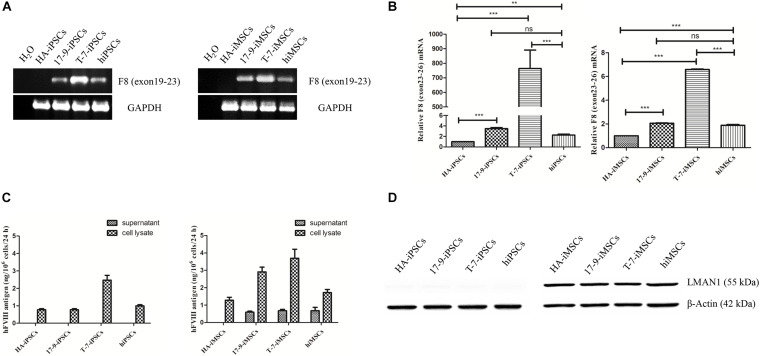
Detection of FVIII expression in iPSCs and iMSCs. **(A)** RT-PCR analysis of *F8* mRNA in iPSCs and iMSCs. Primers were based on exon 19 and exon 23 sequences. *GAPDH* was used as a loading control. **(B)** qRT-PCR analysis of relative *F8* (exons 23–26) mRNA levels. The primers were based on exon 23 and exon 26 sequences. *GAPDH* was used as the internal reference. Bars represent the mean ± SEM (*n* = 3, each group); ***p < 0.001, and **p < 0.01 compared with HA-iPSCs, ns, not significant, and ***p < 0.001 compared with hiPSCs; ***p < 0.001 compared with HA-iMSCs; ns, not significant; and ***p < 0.001 compared with hiMSCs (one-way ANOVA). **(C)** FVIII antigen in iPSCs and iMSCs was detected by ELISA. Bars represent the mean ± SEM (*n* = 3, independent cultures). **(D)** Western blot analysis of LMAN1 protein in iPSCs and iMSCs. β-Actin was used as the internal reference.

Next, the expression of *F8* was detected in iPSCs and iMSCs *via* RT-PCR. Transcripts containing the boundary of exons 22–23 were detected in 17-9-iPSCs, T-7-iPSCs, and hiPSCs and in the corresponding derived 17-9-iMSCs, T-7-iMSCs, and hiMSCs; however, the signal was not detected in HA-iPSCs and HA-iMSCs (Figure 2A), indicating that *F8* was corrected in *F8*-modified iPSCs and iMSCs. The transcription levels of *F8* mRNA (containing exons 23–26) in modified iPSCs (17-9-iPSCs and T-7-iPSCs) and corresponding derived iMSCs (17-9-iMSCs and T-7-iMSCs) were significantly higher than those in HA-iPSCs and HA-iMSCs and similar to or even higher than those in hiPSCs and hiMSCs (Figure 2B).

To evaluate the FVIII expression in iPSCs and iMSCs, the FVIII antigen in the cell lysate and supernatants of iPSCs and iMSCs was detected by ELISA. FVIII expression was detected in all iPSC cell lysates but was not detected in the supernatants, indicating that FVIII was not secreted at the iPSC stage (Figure 2C). In the iMSC stage, ELISA results showed that the FVIII protein levels were 0.59 ± 0.11 ng/(10^6^ at 24 h) and 0.68 ± 0.14 ng/(10^6^ at 24 h) in the supernatant of 17-9-iMSCs and T-7-iMSCs, respectively, but were not detected in the samples of HA-iMSCs. Several studies have demonstrated that the complex formed by lectin mannose-binding 1 (LMAN1) and multiple coagulation factor deficiency 2 (MCFD2) serves as a cargo receptor for efficient transportation of FVIII from the endoplasmic reticulum to the Golgi (Zheng et al., 2010; Zhu et al., 2018). Detection of LMAN1 by western blot indicated that LMAN1 was expressed in HA-iMSCs, 17-9-iMSCs, T-7-iMSCs, and hiMSCs but was barely detectable at the iPSC stage (Figure 2D). These data indicated that 17-9-iMSCs, T-7-iMSCs, and hiMSCs synthesize and secrete FVIII.

### Restoration of FVIII Function in HA Mice by Transplantation of *F8*-Modified iPSC-Derived iMSCs

**FIGURE 3:**
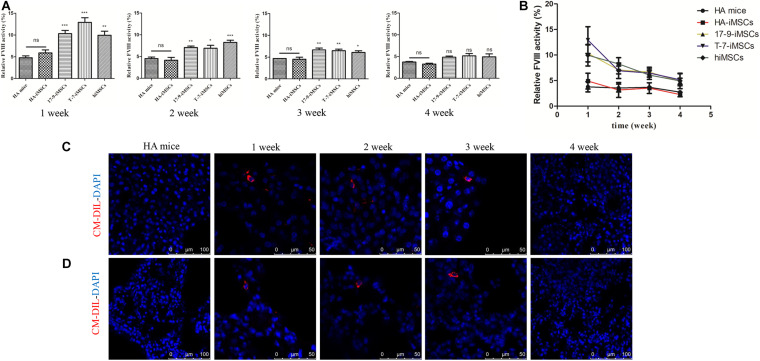
Restoration of FVIII function in HA mice using *F8*-modified iPSC-derived iMSCs. **(A)** Relative FVIII activity was detected 1–4 weeks after transplantation. HA mice, untreated HA mice; HA-iMSCs, HA mice transplanted with HA-iMSCs; 17-9-iMSCs, HA mice transplanted with 17-9-iMSCs; T-7-iMSCs, HA mice transplanted with T-7-iMSCs; hiMSCs, HA mice transplanted with hiMSCs. Bars represent the mean ± SEM, *n* = 5–6; ns, not significant, ***p < 0.001, **p < 0.01, and *p < 0.05 compared with HA mice (one-way ANOVA). **(B)** The data in **(A)** are shown as a line graph to describe the changes in FVIII activity during the 4-week observation period. **(C)** Liver and **(D)** lung tissue sections of iMSC-treated HA mice were analyzed using immunofluorescence; red fluorescence represents CM-Dil-positive cells, and DAPI was used for nuclear staining.

To test whether iMSCs derived from *F8*-modified iPSCs restore FVIII function *in vivo*, we carried out animal studies in HA mice with a disrupted *F8* gene. Initially, we labeled iMSCs using CellTracker^TM^ CM-Dil, a fluorescent dye (Supplementary Figure 2A). Then, CM-Dil-labeled HA-iMSCs, 17-9-iMSCs, T-7-iMSCs, and hiMSCs were infused into HA mice *via* the orbital vein. We collected mouse plasma and assayed FVIII activity at four time points: 1, 2, 3, and 4 weeks after transplantation. The FVIII activity was recalculated as a percentage of the activity in wild-type mice. As shown in Figure 3A, 1 week after transplantation, the FVIII activity in HA mice treated with 17-9-iMSCs, T-7-iMSCs, and hiMSCs reached the highest levels of up to 10.32, 12.91, and 9.96%, respectively, which were significantly higher than that in HA mice (4.78%). From 2 to 3 weeks after transplantation, the FVIII activity in HA mice treated with 17-9-iMSCs, T-7-iMSCs, and hiMSCs was gradually decreased but remained significantly higher than that in HA mice. The changes in FVIII activity during the 4-week observation period are shown in Figure 3B. The data indicate that the FVIII activity in HA mice treated with 17-9-iMSCs, T-7-iMSCs, and hiMSCs was the lowest 4 weeks after transplantation, which was similar to that in HA mice.

Major organs (heart, liver, spleen, lung, and kidney) from treated and HA mice were sectioned to detect CM-Dil-labeled cells by immunofluorescence analysis. CM-Dil-positive cells were observed in the liver (Figure 3C) and lung sections (Figure 3D) of mice treated with iMSCs 1, 2, and 3 weeks after transplantation but were not detected in treated mice 4 weeks after transplantation or in HA mice. CM-Dil-positive cells were not observed in other organs (heart, spleen, and kidney) of treated and HA mice (Supplementary Figure 2B). The data show that iMSCs underwent sustained implantation in some organs, such as the liver and lungs, during the 3-week observation period, indicating that *F8*-modified iPSC-derived iMSCs are likely to restore FVIII function *in vivo*.

### Phenotypic Rescue in HA Mice 1 Week After Transplantation of *F8*-Modified iPSC-Derived iMSCs

**FIGURE 4:**
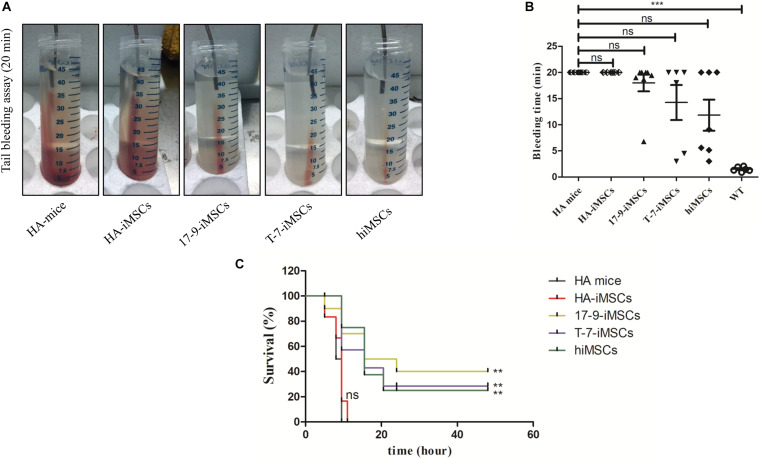
Tail-bleeding assay. **(A)** Representative images of the tails after 20 min of the bleeding assay for each group. **(B)** Bleeding time. WT, C57BL/6 mice. ns, not significant, ***p < 0.001 compared with HA mice (one-way ANOVA). **(C)** Proportions of surviving mice after the tail-clip challenge. HA mice, untreated HA mice (*n* = 6); HA-iMSCs, HA mice transplanted with HA-iMSCs (*n* = 6); 17-9-iMSCs, HA mice transplanted with 17-9-iMSCs (*n* = 8); T-7-iMSCs, HA mice transplanted with T-7-iMSCs (*n* = 6); hiMSCs, HA mice transplanted with hiMSCs (*n* = 7). **p < 0.01 compared with HA mice group (log-rank test).

A tail-bleeding assay was performed to determine the phenotypic rescue in HA mice 1 week after transplantation of iMSCs derived from *F8*-modified iPSCs. Figure 4A shows representative images of the tails after 20 min of the bleeding assay in each group. At the recorded bleeding time, some HA mice engrafted with 17-9-iMSCs (*n* = 8), T-7-iMSCs (*n* = 6), and hiMSCs (*n* = 7) had already developed coagulation, while all mice of the HA group (*n* = 6) and animals treated with HA-iMSCs (*n* = 6) were still bleeding (Figure 4B). We examined the survival rates of each group 48 h after the tail-clip challenge experiment. The data indicated that none of the HA mice or animals receiving HA-iMSCs survived more than 20 h after the tail-clip challenge. Notably, two of the eight HA mice treated with 17-9-iMSCs, one of the six HA mice treated with T-7-iMSCs, and one of the seven HA mice treated with hiMSCs were alive 2 days after the tail-clip challenge, which was the endpoint of this experiment (Figure 4C). These results suggest that transplantation of *F8*-modified iMSCs can partially rescue the bleeding phenotype of HA mice.

## Discussion

Mesenchymal stem cells -based therapy for various diseases has been in progress in clinical trials, including cardiovascular diseases (Zhao et al., 2015; Bartolucci et al., 2017; Butler et al., 2017; Kim et al., 2018), neurodegenerative diseases (Syková et al., 2017; Oh et al., 2018), and autoimmune diseases (Ghoryani et al., 2019), which emphasizes the therapeutic effects of MSCs. Additionally, MSCs have been applied for tissue repair and regeneration (Tan et al., 2016; Gjerde et al., 2018; Sha et al., 2019). Therefore, MSC-based therapy has been proven to be a safe and effective strategy. The immunosuppressive effects with low immunogenicity and the ability to produce endogenous FVIII (Sanada et al., 2013) suggest that MSCs are suitable for HA therapy.

Currently, MSCs used in preclinical studies and clinical trials are mainly isolated from bone marrow and adipose tissue; these sources may be limited in a single donor and may cause heterogeneity (Phinney et al., 1999; Pevsner-Fischer et al., 2011; Zaim et al., 2012; McLeod and Mauck, 2017; Huang et al., 2019). Moreover, the MSC isolation procedure is traumatic for the patients. Therefore, considering that invasive biopsy should be avoided for bleeding diseases, iPSCs generated from urine cells were selected as the source of iMSCs of HA patients. On the one hand, the source of iMSCs can be infinite because iPSCs possess unlimited proliferative capacity (Takahashi et al., 2007; Yu et al., 2007). On the other hand, iMSCs derived from iPSCs can maintain uniform quantity and quality thus avoiding heterogeneity. At present, genetic modification of MSCs is mainly performed *via* viral vector transduction; hence, immunogenicity of the viral capsid and the risk of random insertional mutations has to be addressed (Kotterman et al., 2015; Evens et al., 2018; Milone and O’Doherty, 2018; Shirley et al., 2020). Non-viral genetic modification by targeted integration can be efficiently achieved at the iPSC stage for sustainable expression of the target gene, representing an applicable strategy of *ex vivo* gene therapy for HA. In our study, 17-9-iPSCs and T-7-iPSCs with highly effective FVIII expression and no detectable off-target effects were obtained by gene editing and were selected *in vitro*. This screening strategy based on non-viral transduction may improve safety of subsequent *in vivo* experiments. To our knowledge, this is the first report that uses iMSCs derived from *F8*-modified iPSCs for the treatment of HA mice.

In the present study, the therapeutic level of plasma FVIII activity was sustained for 3 weeks in HA mice transplanted with 17-9-iMSCs, T-7-iMSCs, and hiMSCs. Then, FVIII activity was decreased down to the lowest value at week 4 after transplantation. This decrease may be due to the development of immunogenicity against human cells because we used immunocompetent HA mice that were not treated with immunosuppressive drugs. The SCID-HA mice transplanted with human MSCs presented restored hemostasis 1 week after the injection (Follenzi et al., 2012; Neumeyer et al., 2019). Gao et al. (2019) reported that subcutaneous injection of endothelial colony-forming cells and placenta-derived mesenchymal stromal cells transduced with a BDD-FVIII-expressing lentiviral vector achieved long-term engraftment and FVIII expression for 2 weeks in HA mice treated with cyclosporine A. However, side effects of immunosuppressive drugs have been described (Dobbels et al., 2008; Schmidt et al., 2019; Brami et al., 2020). Wang et al. (2013) implanted hFVIIIBD-MSCs isolated from *hFVIIIBD*-transgenic mice into HA mice by intravenous injection and achieved a therapeutic effect that lasted for 5 weeks. This effect may be attributed to reduced immune rejection between allogeneic inbred mice. In the present study, to some extent, a decrease in CM-Dil-positive iMSCs in the liver and lung sections can explain a decline in plasma FVIII activity in the treated mice, which might have produced hFVIII inhibitors; however, additional verification of this hypothesis is needed. Future studies will involve SCID-HA mice to reduce the immune response and provide a more accurate model of the effect of autologous treatment.

In summary, transplantation of iMSCs derived from *F8*-corrected patient iPSCs into HA mice *via* the orbital vein restored FVIII function and rescued the HA phenotype. Our results provide proof-of-concept for cell-based gene therapy *via* targeted genetic correction of FVIII in autologous iPSCs. In future studies, iMSCs will be transplanted into an HA mouse model with immunodeficiency to test long-term efficacy of the treatment. This work also suggests a feasible strategy of therapeutic gene editing for HA and other genetic birth defects involving large sequence variants.

## Data Availability Statement

The original contributions presented in the study are included in the article/Supplementary Material, further inquiries can be directed to the corresponding author/s.

## Ethics Statement

The animal study was reviewed and approved by School of Life Sciences of Central South University.

## Author Contributions

LW and ZH designed the research and edited the manuscript. LQ and MX performed the experiments. LQ prepared the figures and drafted the manuscript. MZ edited the manuscript. XL assisted in manuscript preparation. All authors read and approved the final manuscript.

## Conflict of Interest

The authors declare that the research was conducted in the absence of any commercial or financial relationships that could be construed as a potential conflict of interest.
