# Supplementary material for: Restoration of FVIII Function and Phenotypic Rescue in Hemophilia A Mice by Transplantation of MSCs Derived From F8-Modified iPSCs
Source: Front Cell Dev Biol. 2021 Feb 11;9:630353. doi: 10.3389/fcell.2021.630353 (PMC7905062; doi:10.3389/fcell.2021.630353)
Supplement: Supplementary file 1 [file Data_Sheet_1.DOCX]

Supplementary Material

**Supplementary Figure 1.**

**(A)**


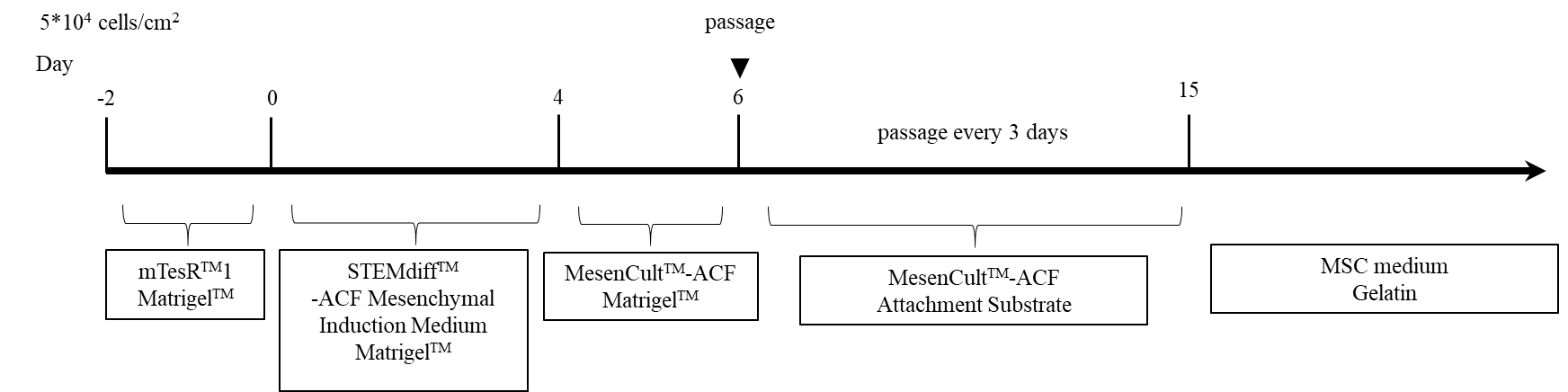


**(B)**


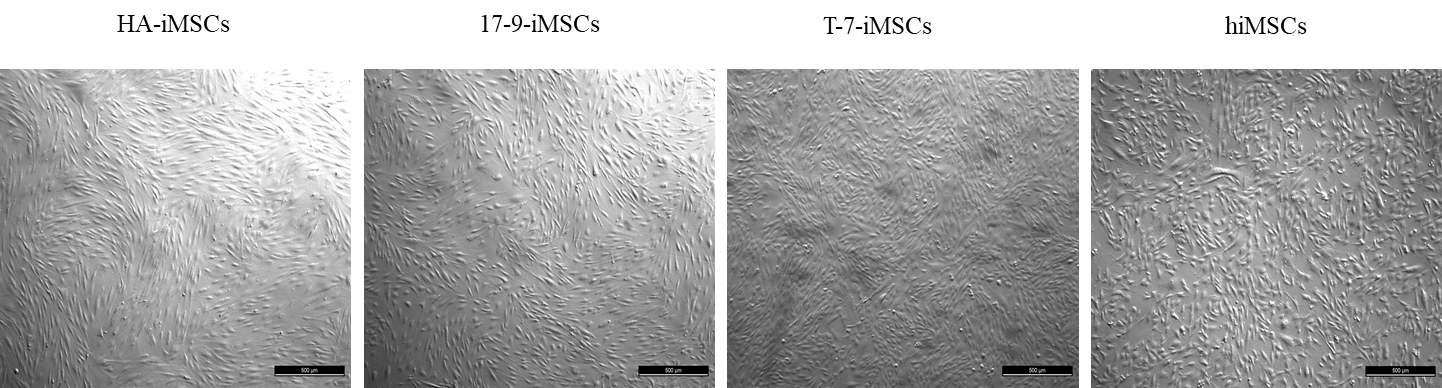


**Supplementary Figure 1.** **Differentiation of iPSCs into iMSCs. (A)** Flow chart of the modified protocol for differentiation of iPSCs into iMSCs. **(B)** Morphology of the iMSCs derived from HA-iPSCs, 17-9-iPSCs, T-7-iPSCs, and hiPSCs at passage 5.

**Supplementary Figure 2.**

**(A)**


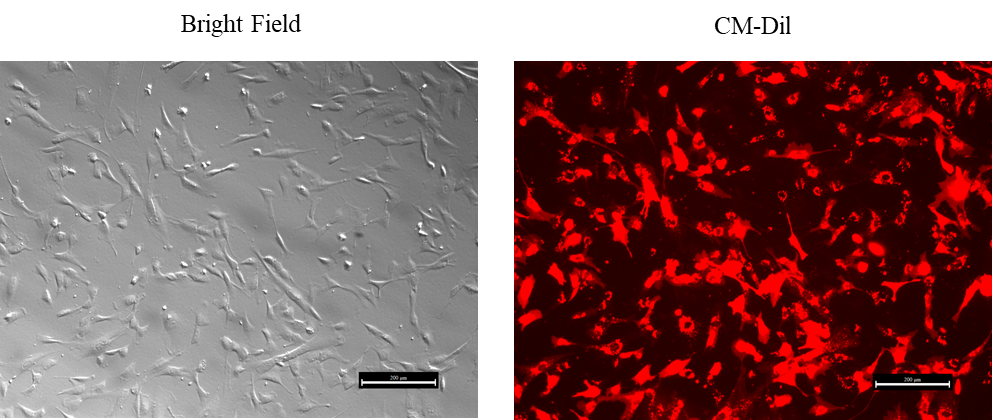


**(B)**


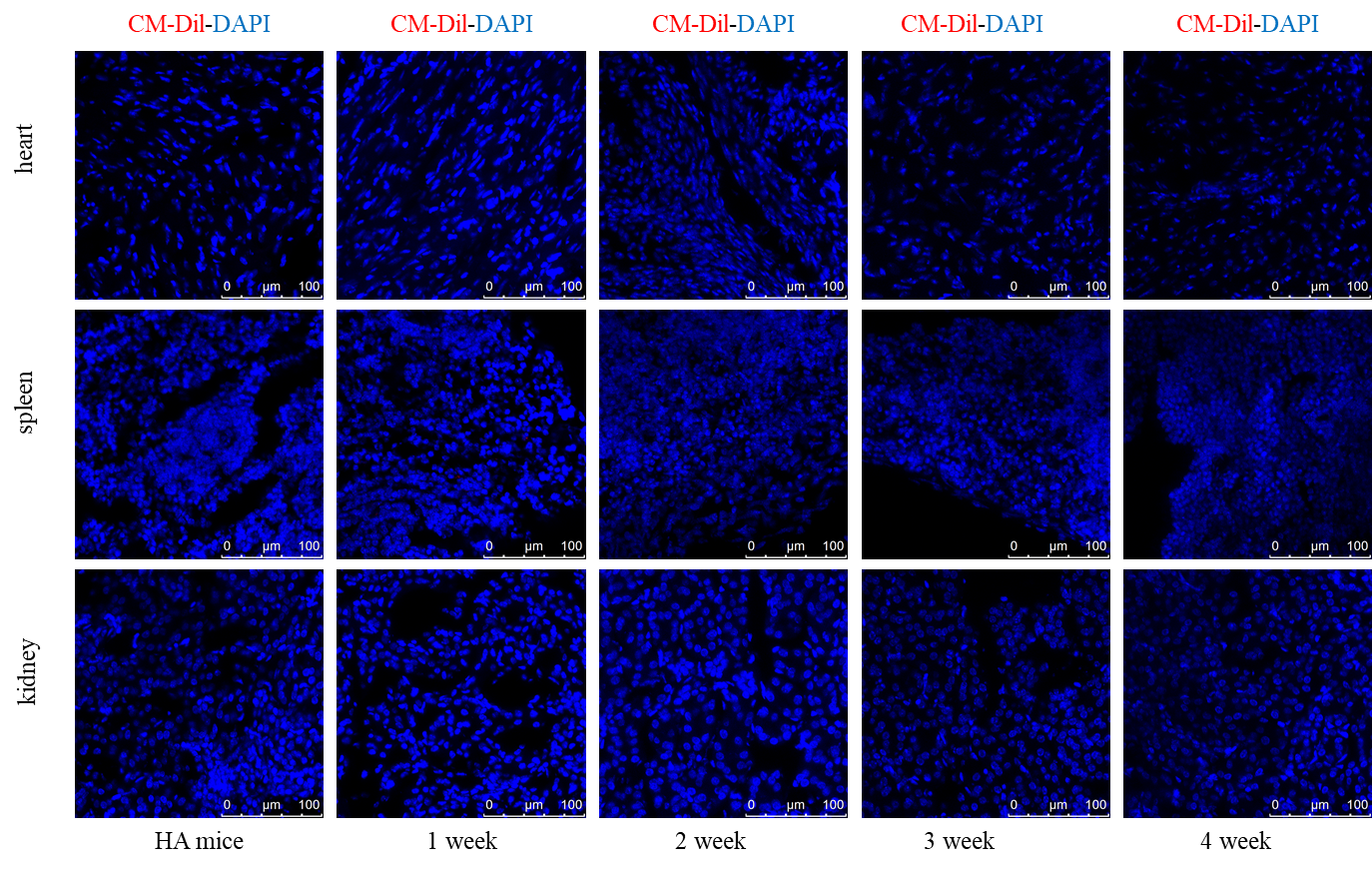


**Supplementary Figure 2.** **Transplantation of iMSCs into HA mice. (A)** The iMSCs were labeled with CM-Dil before transplantation into HA mice. The red fluorescence showed the CM-Dil labeled cells. **(B)** Heart, spleen, and kidney tissue sections of iMSCs-transplanted HA mice were analyzed using immunofluorescence; Red fluorescence represent CM-Dil-positive cells, DAPI was used for nuclear staining. HA mice represent non-transplanted HA mice, 1-4 week represent the timepoints during the whole observation.

**Supplementary Tables.**

**Table S1. Primers used in RT-PCR and qRT-PCR, related to Figure 2.**

| **Primer name** | **Sequence (5^’^ to 3^’^)** | **Used for the experiment of** |
| --- | --- | --- |
| F8-RT-E19F | GCTGGGATGAGCACACTTTT | RT-PCR for F8-E19-23 |
| F8-RT-E23R | TCAACTCCATGCGAAGAGTG |  |
| F8-RT-E23F | CACTCTTCGCATGGAGTTGA | qRT-PCR for F8-E23-26 |
| F8-RT-E26R | GGGGGTGAATTCGAAGGTAG |  |
| GAPDH-F | GGGGAGCCAAAAGGGTCATCATCT | RT-PCR and qRT-PCR for GAPDH |
| GAPDH-R | GACGCCTGCTTCACCACCTTCTTG |  |
